# Laparoscopic adenomyomectomy combined with levonorgestrel-releasing intrauterine system is effective for long-term management of adenomyosis

**DOI:** 10.1186/s12905-023-02795-1

**Published:** 2024-01-08

**Authors:** Jilan Jiang, Yilian Pan, Jin Yu, Ye Zhang, Yeping Yang, Hong Xu, Feng Sun

**Affiliations:** 1 Department of Gynecology & Obstetrics, International Peace Maternity & Child Health Hospital, Shanghai Jiao Tong University School of Medicine, No.910 Hengshan Road, Xuhui District, Shanghai, China; 2 Shanghai Key Laboratory of Embryo Original Diseases, Shanghai, China; 3 Shanghai Municipal Key Clinical Speciality, Shanghai, China

**Keywords:** Adenomyosis, Laparoscopic adenomyomectomy, Levonorgestrel-releasing intrauterine system, Recurrence

## Abstract

**Backgroud:**

Laparoscopic adenomyomectomy combined with intraoperative placement of levonorgestrel-releasing intrauterine device (LNG-IUS) is a novel conservative surgical procedure for adenomyosis. Our study aimed to compare the efficacy of surgery with or without intraoperative placement of LNG-IUS treatment in adenomyosis.

**Methods:**

We retrospectively reviewed the medical records of adenomyosis patients who received laparoscopic adenomyomectomy from January 2014 to April 2020, finally including 70 patients undergoing surgery-LNG-IUS as group A and 69 patients undegoing surgery only as group B. Risk factors for three-year relapse were analyzed using Cox’s multivariate proportional hazard analysis.

**Results:**

Visual analog scale and Mansfield-Voda-Jorgensen Menstrual Bleeding Scale scores of group A at 3, 6, 12, 24, and 36 months were significantly lower than those of group B at the corresponding points (*P* < .001 for both scales). Individuals in both groups showed statistically significant symptom relief. The recurrence rate in group A was significantly lower than that in group B at 36 months after the surgery (2.94% vs. 32.84%, *P < *.001). A cox proportional hazard model showed that relapse was significantly associated with coexisting ovarian endometriosis (adjusted hazard ratio [aHR], 2.94; 95% confidence interval [CI], 1.33–7.02, *P =* .015). Patients who received surgery-LNG-IUS had a lower risk of recurrence than those with surgery-alone (aHR, 0.07; 95% CI, 0.016–0.31, *P* < .001).

**Conclusions:**

Conservative surgery with intraoperative placement of LNG-IUS is effective and well-accepted for long-term therapy with a lower recurrence rate for adenomyosis. Coexistent ovarian endometriosis is a major factor for adenomyosis relapse.

## Background

Uterine adenomyosis is described as a benign gynecological disease featured by aberrant development of endometrial glands and stroma within the myometrium [1]. Diffuse and/ or focal lesions can occur in the internal or external layers of the myometrium [2]. Clinical symptoms associated with adenomyosis include menorrhagia, dysmenorrhea, and an enlarged uterus [3]. Because an increasing number of women choose to preserve their uteri, conventional hysterectomy has become less acceptable as treatment for adenomyosis.

Various treatment strategies can be used for adenomyosis [4]. Drug therapy, such as nonsteroidal anti-inflammatory drugs, oral contraceptives, gonadotropin-releasing hormone agonists (GnRH-a), and progestins can all be used for symptom relief. However, once these treatments stop, symptoms will soon reoccur. A surgical approach for adenomyosis may be considered when medical management fails, either due to breakthrough of pain or intolerable side effects from drug therapy or the patient wants a definitive diagnosis [5]. Laparoscopic adenomyomectomy has been increasingly performed worldwide and has been demonstrated to be a safe and effective therapeutic modality [6].

It is reported that, after conservative surgery, over three-fourths of patients achieved complete relief, and the recurrence rate of symptoms is about 9% after the complete excision [7]. As conservative surgery for adenomyosis cannot remove adenomyotic focus thoroughly, even if adenomyosis is characterized by focal lesions, adenomyosis recurrence is unavoidable thus the efficacy of adenomyomectomy decreases over time after surgery. There are still some patients suffering from symptoms relapsed within 1 year of surgery.

By releasing levonorgestrel locally, LNG-IUS exerts progesterone-like effect on the endometrium, which then relieves dysmenorrhea and reduces menstrual flow [8]. The LNG-IUS is a suitable alternative to surgery for the management of dysmenorrhea. However, in patients with large adenomyosis, the LNG-IUS has a high expulsion rate (37.5%) [9]. Lee et al. reported the LNG-IUD expulsion rate increased significantly when the uterine volume was greater than 150 ml [10]. Laparoscopic adenomyomectomy reduces the size of the uterus by removing the lesions, thus creating appropriate conditions for LNG-IUS placement.

Previously, we reported that the combination of laparoscopic adenomyomectomy with LNG-IUS is an effective and novel conservative surgical procedure for adenomyosis [11]. However, the symptom recurrence rate associated with this modality has not yet been reported. Therefore, we conducted this retrospective study to compare the efficacy of surgery alone with combined surgical-LNG-IUS treatment in adenomyosis and to explore the risk factors for symptom recurrence.

## Methods

### Study design and population

This retrospectively study between January 2014 and April 2020 has been approved by the ethic committee of the International Peace Maternity and Child Health Hospital of the China Welfare Institute (GKLW 2017-71). For the adenomyosis patients who wanted uterus-sparing sugery without fertility desire, LNG-IUS placement was recommended in the surgery. The enrollment criteria included: (1) age between 20 and 48 years, (2) severe dysmenorrhea and/or menorrhagia, (3) availability for transvaginal ultrasound examination data, and (4) postoperative histopathological confirmation of adenomyosis. The exclusion criteria included: (1) submucous myoma, (2) breast cancer, (3) pathologic discoveries of malignancy (e.g., endometrial cancer), (4) previous surgery for adenomyosis, (5) GnRH agonist therapy, or other hormone therapies after surgery.

### Data collection and definition

A scale ranging from 1 to 6 was used to assess menstrual blood loss according to the Mansfield–Voda–Jorgensen menstrual bleeding scale (MVJ). Menorrhagia is defined as the MVJ score ≥ 5 [12]. The degree of menstrual pain was evaluated by visual analog scale (VAS) [13]. And transvaginal ultrasonography was applied to measure the uterinevolume by the formula: volume = 0.5233 × D1 × D2 × D3, where D1, D2, D3 represented the longitudinal dimension, anteroposterior dimension, and the transverse dimension, respectively [14]. On ultrasonography, the extent of an adenomyosis was determined by its maximum diameter. A hemoglobin level of < 110 g/L was defined as anemia. The serum carbohydrate antigen 125 (CA125) levels were determined using a sandwich ELISA kit (R&D Systems, Minneapolis, MN, USA) according to the manufacturer’s instructions.

Because there was no uniform agreement on the best long-term therapeutic method to prevent recurrences of adenomyosis, these women who opt for laparoscopic adenomyomectomy were informed that they could choose any one of the following therapies, depending on their willingness and preferences. Therapies included adenomyomectomy with LNG-IUS, GnRH-a postoperatively, gestrinone post-surgically, and and pure follow-up. To verify, laparoscopic adenomyomectomy with LNG-IUS, if chosen by patients, was notified that if the depth was < 10 cm, we inserted an LNG-IUS (Mirena, Bayer, Shanghai, China) containing 52 mg of levonorgestrel immediately after the completion of the operation, and if not, we did not insert it. The surgical procedure and methods of laparoscopic adenomyomectomy were the same as the previous study in our center [11]. Through the hospital electronic record system, we collected the demographics of the enrolled patients and conducted a telephone interview to obtain the additional data 3 years after the surgery. Symptom relapse included dysmenorrhea or menorrhagia reoccur, which was defined as when VAS increased by a threshold of 3 and by participants asking for other medical treatments for symptom relief or the MVJ score ≥ 5 [2, 15].

### Statistical analysis

For continuous variables with Gaussian distributions, mean ± standard deviation is used, or mean is used with its 95%CI. Categorical variables were expressed as numbers/ categories. Parametric continuous variables were compared using Student’s t-test, while nonparametric variables using the Wilcoxon rank-sum test. The chi-squared test was used to compare categorical variables. Cox’s multivariate proportional hazard analyses were performed to identify independent correlates between potential confounding factors (univariate *P* < .2). The results are reported as aHR with 95% CI. Significance was assumed when *P* was < .05. We used SPSS 26.0.0 for all analyses (SPSS, Inc. Chicago, IL, USA).

## Results

**Fig. 1:**
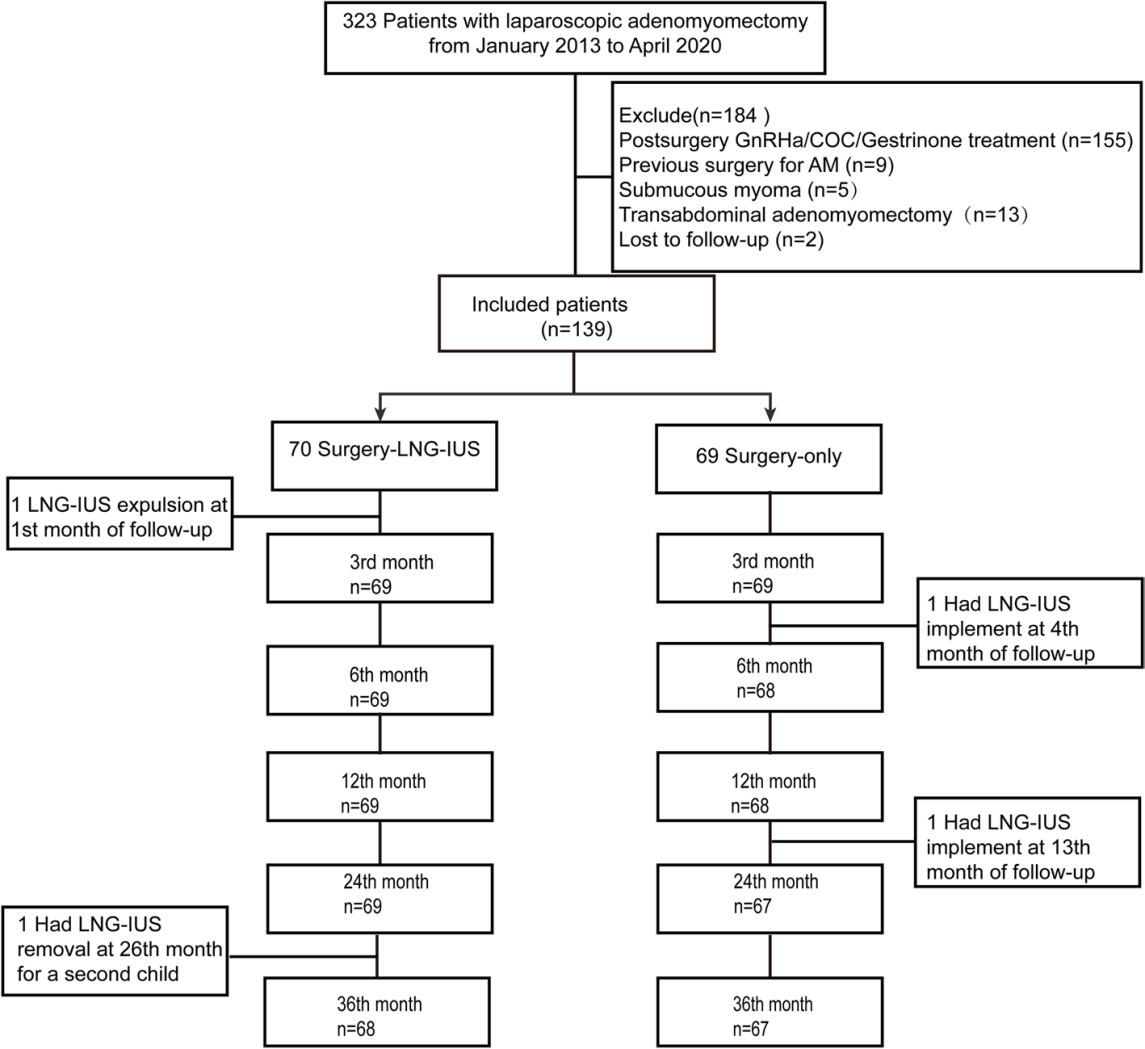
Flowchart of this study Abbreviation: COC, combined oral contraceptive; GnRH, gonadotropin-releasing hormone; LNG-IUS, levonorgestrel-releasing intrauterine device

We initially identified 323 patients who received laparoscopic adenomyomectomy with histopathological tissue samples confirming diagnoses. However, 155 patients had GnRH-a/COC/Gestrinone therapy, 13 took transabdominal adenomyomectomy, 9 took surgeries for adenomyosis previously and 5 had submucous myoma; all were excluded. Besides, 2 patients lost follow up after surgery. In the end, 70 patients undergoing laparoscopic adenomyomectomy combined with intraoperative insertion of LNG-IUS were included in group A and 69 patients undergoing laparoscopic adenomyomectomy only were included in group B (Fig. 1). And all those 139 women do not have perimenopausal or menopausal symptom at follow-up. In group A, one patient had LNG-IUS expulsion one month after the surgery and another one removed the LNG-IUS preparing for a second child. In group B, two women had LNG-IUS to prevent adenomyosis relapse and contraception, on the 4th and 13th month after the surgery, respectively.

Patient characteristics are presented in Table 1. No significant differences in age, body mass index (BMI), uterine volume, maximum diameters of adenomyosis lesions, parity, previous abortions, previous abdominal surgery, hemoglobin levels, CA125, VAS scores, or menorrhagia were found between the two groups (all *P* > .05, Table 1).

**Table 1:** Baseline characteristics of two groups

	Group A (*n* = 70)	Group B (*n* = 69)	*P*
Age (years)	39.4 (38.4–40.4)	39.5 (38.2–40.8)	.576
BMI (kg/ m^2)	22.9 ± 2.92	22.6 ± 2.28	.418
Uterine volume (cm^3)	189 0.1(171.1-207.2)	178.2 (158.8-197.6)	.204
Maximum diameter (cm)	56.5 (53.5–59.5)	55.0 (52.3–57.8)	.549
Nullipara, n(%)	9 (12.9%)	13 (18.8%)	.352
Multipara, n(%)	61 (87.1%)	56 (81.2%)	
Number of abortions, n	1.4 (1.1–1.8)	1.3 (0.9–1.7)	.460
Previous abdominal surgery, n(%)			
Cesarean section	27 (38.6%)	34 (49.3%)	.204
Myomectomy	1 (1.4%)	1 (1.4%)	>.99
Appendectomy	1 (1.4%)	0	>.99
Adnexal surgery	4 (5.7%)	8 (11.6%)	.243
Preoperative hemoglobin, g/dL	115.1 ± 13.3	116.6 ± 13.5	.487
Preoperative CA125, U/ml	138.5 (106.6-170.4)	104.3 (84.8-123.9)	.311
Preoperative VAS score	7.7 (7.2–8.2)	7.1 (6.5–7.8)	.150
Preoperative MVJ score	4.7 (4.4-5.0)	4.5 (4.2–4.8)	.292
MVJ ≥ 5, n %	47 (67.1%)	40 (58.0%)	.296
Severe Dysmenorrhea (VAS ≥ 7), n %	58 (82.8%)	56 ( 81.2%)	.794
Follow-up period,month	62.9 (58.9–66.9)	56.2 (50.8–61.7)	
Follow-up range,month	24, 101	24, 141	

*Abbreviation:* *BMI* Body mass index (calculated as weight in kilograms divided by the square of height in meters), *CA125* Carbohydrate antigen 125, *VAS* Visual analog scale

The operative findings between the two groups are shown in Table 2. None of the women required conversion to laparotomy. The operative time in group A was significantly longer than in group B (180.1, 95%CI, 168.5-191.7 vs. 156.5, 95%CI, 142.9-170.0 min, *P* = .002). An analysis of blood loss, specimen weight, hospital stay, and postoperative hemoglobin revealed no significant differences. One patient in group A underwent intraoperative blood transfusion for the large adenomyosis leisions. The hemorrhage and blood transfusion volumes were approximately 1000 ml and 400 ml, respectively. No postoperative complications, such as severe infection or intestinal obstruction, were observed in any patient. No significant differences were found in the number of concomitant ovarian endometriosis, deeply infiltrating endometriosis (DIE) or peritoneal endometriosis between the two groups. In group A, one patient had LNG-IUS embedment when changed the LNG-IUS five years after the surgery, 4 patients reported weight gain, and 1 reported acne during follow-up.

**Table 2:** Operation findings and complications in two groups

Variables	Group A (*n* = 70)	Group B (*n* = 69)	*P*
Operation time (min)	180.1 (168.5-191.7)	156.5 (142.9–170.0)	.002
Blood loss (ml)	177.43 (141.0-213.9)	197.1 (161.1-233.1)	.300
Postoperative hemoglobin D1, g/dL	107.2 ± 10.6	107.1 ± 14.1	.992
Concomitant OEM	13 (18.6%)	14 (20.3%)	.798
Concomitant DIE	18 (25.7%)	14 (20.3%)	.448
Concomitant PEM	10 (14.3%)	6 (8.7%)	.302
Hospital stay (days)	8.3 (7.8–8.7)	8.0 (7.5–8.4)	.740
Conversion to laparotomy	0	0	
Postoperative complications	0	0	
Intraoperative blood transfusion, n (%)	1 (1.4%)	0	> .99
IUS expulsion, n	1 (1.4%)	-	
Acne, n	1(1.4%)	-	
Weigh gain, n	4(5.7%)	-	
IUS embedment, n	1 (1.4%)	-	

*Abbreviation:* *DIE* Deep invasive endometriosis, *OME* Ovarian endometrium, *PEM* Peritoneal endometriosis, Postoperative hemoglobin D1, on the first postoperative day

The changes in VAS scores, MVJ scores, and recurrence rates are shown in Table 3. Pre-surgery VAS scores of 7.7 (7.2–8.2) and 7.1 (6.5–7.8) fell to 0.21 (0.05–0.38) and 0.81 (0.52–1.10), respectively, at three month follow-up in Groups A and B, respectively, and then remained lower levels at 6, 12, 24, 36 month. The differences in VAS scores between pre-surgery and subsequent follow-up scores at 3, 6, 12, 24, and 36 months were all statistically significant (*P* < .001). The VAS scores were significantly lower in group A than in group B at 3, 6, 12, 24, and 36 months after surgery (*P* < .001).

**Table 3:** Mean differences in VAS score, MVJ score, and recurrence rate after surgery

	Group A	Group B	*P*^a^	*P*^b^	*P*^c^
**3rd month**	*n* = 69	*n* = 69			
VAS	0.21 (0.05–0.38)	0.81 (0.52–1.10)	<.001	<.001	<.001
MVJ	1.03 (0.82–1.24)	2.41 (2.19–2.63)	<.001	<.001	<.001
Recurrence, n(%)	0	0	*-*		
**6th month**	*n* = 69	*n* = 68			
VAS	0.20 (0.04–0.37)	1.04 (0.69–1.40)	<.001	<.001	<.001
MVJ	1.03 (0.82–1.24)	2.74 (2.47-3.00)	<.001	<.001	<.001
Recurrence, n%	0	3 (4.41%)	.039		
**12th month**	*n* = 69	*n* = 68			
VAS	0.26 (0.05–0.47)	1.39 (0.98–1.79)	<.001	<.001	<.001
MVJ	0.97 (0.77–1.17)	2.70 (2.44–2.96)	<.001	<.001	<.001
Recurrence, n(%)	1 (1.45%)	9 (13.24%)	.009		
**24th month**	*n* = 69	*n* = 67			
VAS	0.35 (0.09–0.60)	1.52 (1.04–2.01)	<.001	<.001	<.001
MVJ	1.04 (0.81–1.27)	2.74 (2.47–3.01)	<.001	<.001	<.001
Recurrence, n(%)	1 (1.45%)	14 (20.90%)	<.001		
**36th month**	*n* = 68	*n* = 67			
VAS	0.47 (0.10–0.84)	1.84 (1.25–2.42)	<.001	<.001	<.001
MVJ	1.09 (0.84–1.34)	2.95 (2.66–3.24)	<.001	<.001	<.001
Recurrence, n(%)	2 (2.94%)	22 (32.84%)	<.001		

*Abbreviation:* *MVJ* Mansfield-Voda-Jorgensen Menstrual Bleeding Scale, *VAS* Visual analog scale

^a^ Group A compared with group B

^b^ Compared with preoperative in group A

^c^ Compared with preoperative in group B

At 3rd-month follow up, the mean MVJ scores for menorrhagia showed a decline from the baseline of 4.7 (4.4-5.0) to 1.03 (0.82–1.24) and remained low at the end of the 36-month follow-up in group A (*P* < .001). The mean MVJ scores for menorrhagia in group B fell from 4.5 (4.2–4.8) to 2.41 (2.19–2.63) and maintained the low level at 36 months (*P* < .001). The mean MVJ scores were significantly lower in group A than in group B at 3, 6, 12, 24, and 36 months after surgery (*P* < .001).

At the 3rd month after the surgery, there was no significant difference in the recurrence rate. But the recurrence rates in group B began to rise significantly from the 6th month on, and recurrence rates in group B were significant higher than Group A, 4.41% vs. 0%, 13.24% vs. 1.45%, 20.90% vs. 1.45% at the 6th month, 12th month, and 24th months after the surgery, respectively. And the cumulative recurrence rate during the 3 years follow-up period was 2/70 (2.86%) in group A and 22/69 (32.88%) in group B, and the difference was significant (*P* < .001). Moreover, a cox proportional hazard model showed that relapse was significantly associated with coexisting ovarian endometriosis (aHR, 2.94; 95%CI, 1.33–7.02, *P =* .015). Patients who received surgery-LNG-IUS had a lower risk of recurrence than those with surgery-alone (aHR, 0.07; 95% CI, 0.016–0.31, *P* <.001) (Table 4).

**Table 4:** Multivariate survival analysis of the association between risk factors and adenomyosis recurrence

	Observation period = 36 months			
Exposure categories	Recurrence, n(%)	HR (95%CI)	aHR (95%CI)	*P*
**Sugery-LNG-IUS**				
No	22 (31.88%)	Ref	Ref	
Yes	2 (2.86%)	0.076(0.018–0.322)	0.07(0.016–0.31)	< .001
**Concomitant OEM**				
No	13(11.61%)	Ref	Ref	
Yes	11(40.74%)	3.98(1.78–8.89)	2.94(1.33–7.02)	.015
Preoperative CA125, U/ml	-	1.00(0.99–1.01)	1.00(0.99–1.01)	.099

*Abbreviation:* *aHR* Adjusted hazards ratio, *LNG-IUS* Levonorgestrel-releasing intrauterine device, *OEM* Ovarian endometrium, *VAS* Visual analog scale

The overall recurrence rate was 24/139 (17.27%) shown in Table 3. Among those relapsed patients, one had hysterectomy, four had LNG-IUS implement and 12 took Dienogest/ COC treatment. And three recurrent women took non-steroid anti-inflammatory drugs and two took traditional Chinese medicine.

## Discussion

In this retrospective study, which included 139 women with adenomyosis, we found that laparoscopic adenomyomectomy effectively relieved the severity of the adenomyosis symptoms, as assessed by the VAS and MVJ scores. But surgery combined LNG-IUS was more effective than surgery-only in the treatment of adenomyosis caused symptoms, such as menorrhagia and dysmenorrhea. Furthermore, it was obvious that surgery- LNG-IUS in preventing recurrence was superior than surgery-only patients in the management of adenomyosis after surgery therapy.

For the women suffered from symptomatic adenomyosis, if they don’t have willingness to have children, the conventional treatment is hysterectomy. There is, however, an increase in the number of women with adenomyosis who wish to retain their uteri because of the recent trend toward organ-preserving surgery and delayed pregnancy. The challenge of treating symptomatic women who want to maintain their fertility is daunting. Medical management can be effective, but it is often transient, and relapse of symptoms and signs almost always occurs once treatment is halted [16]. Conservative surgery is similar to myomectomy, either by laparotomy, laparoscopy [17], or robot-assisted laparoscopy [18]. In a two-year study, more than 90% of patients were satisfied and experienced dramatic improvement in their symptoms after the uterine-sparing surgery [15]. Uterine-sparing surgery for adenomyosis has shown promising results [19]. There is a good chance that severe diffuse uterine adenomyosis can be treated successfully with double-flap laparoscopic adenomyomectomy [20]. All surgeries in our study were performed laparoscopically without conversion to laparotomy. Our study showed that conservative surgery, whether with or without LNG-IUS, effectively relieved the severity of the adenomyosis symptoms, as assessed by the VAS and MVJ scores [21]. But, we found that the surgery-LNG-IUS group was more effective in treating the adenomyosis than the surgery-only group during the 3-year follow-up period. Our study confirms the benefits of surgical-LNG-IUS treatment for adenomyosis symptom improvement, which is in line with previous studies [22]. We also showed that the recurrence rate of adenomyosis after laparoscopic adenomyomecomy was significantly lower in adenomyosis patients with LNG-IUS than that in adenomyosis patients without LNG-IUS, which are consistent with the previous reports of Zhu [20] and Yu [23] et al.

Yu demonstrated that accompanying endometriosis was an independent risk factor for relapse [23] and another study reported that the recurrence rate of adenomyosis in patients with endometriosis was higher than that in patients without endometriosis [20]. Consistent with our recent retrospective study [24], we also found that the coexistence of ovarian endometrioma was associated with higher recurrence. Studies have shown a strong association between adenomyosis and endometriosis [25]. What’s more, endometriosis also causes pelvic pain and its recurrence is very common after conservative surgery, which could lead to increased relapse of menorrhagia. Similar to other studies, there was no significant correlation between the CA125 level and adenomyosis recurrence [20].

Although our study suggests a benefit of intraoperative placement of LNG-IUS in the management of women with symptomatic focal adenomyosis, many limitations could not be avoided. First, this was a retrospective study; thus, it may contain biases with regard to patient characteristics. And the side effects of LNG-IUS, such as breast tenderness, headache and dizziness, depressive mood disorders and pelvic pain were not well recorded. Second, the sample size in each group of patients was small, and the clinical data originated from a single hospital rather than multiple centers. Larger randomized clinical studies are needed to evaluate the clinical usefulness of this treatment and long-term follow-up data are required to confirm our results.

To the best of our knowledge, this is the first study comparing the effectiveness and symptoms recurrence rates of adenomyosis following conservative surgery with or without LNG-IUS intraoperatively. Moreover, based on our three-year follow-up, conservative surgery-LNG-IUS treatment was more effective than surgery alone in controlling symptoms and reducing recurrence rates.

## Conclusion

In conclusion, conservative surgery with intraoperative placement of LNG-IUS is effective and well-accepted for long-term therapy with a lower recurrence rate for adenomyosis. Coexistent ovarian endometriosis is also a major factor for adenomyosis relapse.

## Data Availability

All data generated or analyzed during this study are included in this published article.

## Abbreviations

BMI: Body mass index
CA125: Carbohydrate antigen 125
COC: Combined oral contraceptive
DIE: Deep invasive endometriosis
GnRH: Gonadotropin-releasing hormone
LNG-IUS: Levonorgestrel-releasing intrauterine device
MVJ: Mansfield–Voda–Jorgensen menstrual bleeding scale
VAS: Visual analog scale
